# An Atypical Case of Lactobacillus jensenii Discitis and Osteomyelitis

**DOI:** 10.7759/cureus.98774

**Published:** 2025-12-08

**Authors:** Tarisai D Chiborise, Tarvinder S Gilotra

**Affiliations:** 1 Infectious Diseases, Catholic Health, Kenmore, USA

**Keywords:** lactobacillus bacteria, lactobacillus jensenii, lactobacillus species, vertebral discitis, vertebral osteomyelitis

## Abstract

*Lactobacillus*species are generally considered beneficial and are not typically pathogenic in humans. Bloodstream and tissue infections are uncommon and usually occur in immunocompromised patients. We present a case of a 63-year-old female with *Lactobacillus jensenii* lumbosacral discitis and osteomyelitis, complicated by small bilateral psoas abscesses and myositis. L4-L5 lumbar biopsy confirmed the presence of *L. jensenii* via next-generation sequencing after conventional tissue and blood cultures were negative. The presumed etiology of this infection was a genitourinary source. The patient received piperacillin-tazobactam for six weeks, followed by a transition to amoxicillin-clavulanate as a consolidative regimen, completing a roughly two-month antimicrobial course. Her symptoms, including gait instability and recurrent falls, resolved satisfactorily, with no concerning recurrence after discontinuation of antibiotics.

## Introduction

Vertebral and disc infections are most often associated with Gram-positive cocci and, less frequently, Gram-negative pathogens [1]. The most commonly isolated organisms from tested specimens include *Staphylococcus aureus*, coagulase-negative *Staphylococcus *species, and *Streptococcus *species [2]. Infections may spread hematogenously or contiguously from skin or visceral organ infections [1]. In immunosuppressed patients, less common organisms such as *Mycobacterium tuberculosis*, fungi, *Pseudomonas aeruginosa*, and, rarely, *Lactobacillus *species may be encountered [1,2]. Frequently reported predisposing factors include immunodeficiency states, vertebral surgery, intravenous drug use, intravenous catheter use, diabetes mellitus, infective endocarditis, malignancy, and dental infections or procedures [1,2].

## Case presentation

We present a case of a 63-year-old female who experienced progressive lower back pain and lower extremity weakness over a two-month period, resulting in gait instability and recurrent falls. Examination revealed flaccid paraparesis of the bilateral lower extremities with symmetric hyporeflexia. Bilateral Babinski reflexes and plantar responses were negative.

MRI demonstrated lumbosacral discitis and osteomyelitis, with a paravertebral phlegmonous process including small bilateral psoas abscesses and surrounding myositis. The source of infection was unclear, with no major risk factors identified other than pancytopenia of unclear etiology. Further evaluation of pancytopenia was unrevealing and included negative serologies for human immunodeficiency virus, hepatitis B, hepatitis C, Epstein-Barr virus, and cytomegalovirus. Vitamin B12 and folate levels were normal. There was no evidence of hemolysis, and the peripheral smear revealed no malignant cells. Biopsy staining was negative for acid-fast bacilli and fungal elements. Inflammatory markers were elevated. A transthoracic echocardiogram showed no vegetations.

Her medical history was notable for diabetes mellitus and maxillary dentures. She also reported prolonged vaginal bleeding following new sexual intercourse after many years of abstinence, occurring a few months prior to admission. Her diabetes was poorly controlled and managed with oral hypoglycemic medications; no other regular medications were reported. She was unaware of any active gynecologic infection at the time of presentation and denied recent dental or spinal procedures.

**Figure 1:**
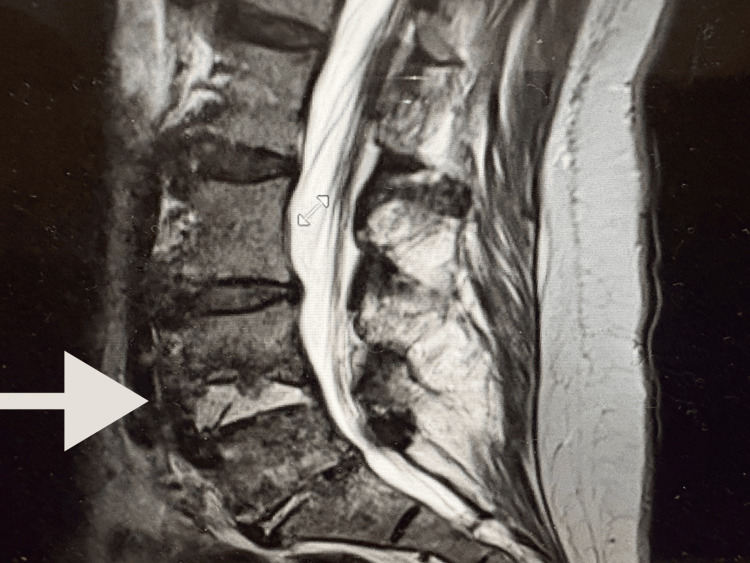
T2-weighted MRI of the lumbar spine showing L4-L5 vertebral osteomyelitis and discitis with Modic type 1 changes, along with a paravertebral phlegmonous process including small bilateral psoas abscesses and surrounding myositis

**Figure 2:**
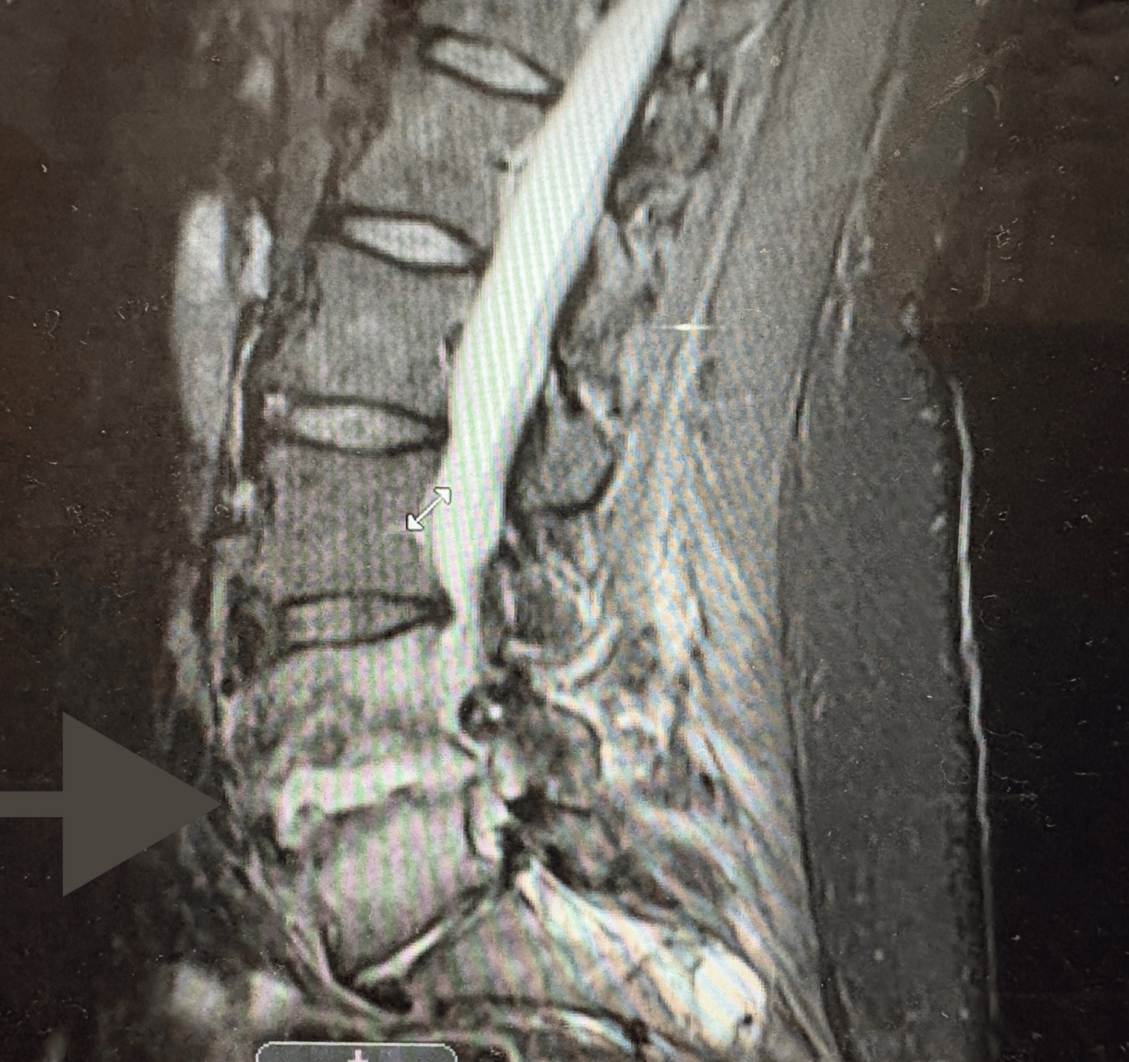
STIR sequence MRI of the lumbar spine at L4-L5 demonstrating vertebral discitis and osteomyelitis, confirming bilateral psoas abscesses with rim enhancement and inflammatory changes extending into the paraspinal musculature STIR, short tau inversion recovery

Urinalysis was positive for bacteriuria, and cultures grew *Escherichia coli*. Admission complete blood count showed leukopenia, anemia, and thrombocytopenia (Table 1). Blood cultures were negative. L4-L5 disc biopsy was positive for *Lactobacillus jensenii *on next-generation sequencing, while conventional tissue cultures were negative (Figure 1, Figure 2).

**Table 1:** Laboratory findings on admission

Parameter	Results	Reference range
Complete blood count		
White blood cell	3.1 × 10⁹/L	4.0-11.0 × 10⁹/L
Neutrophils	67%	40-75%
Red blood cell	2.78 × 10¹²/L	4.0-5.2 × 10¹²/L
Hemoglobin	8.7 g/dL	12.0-15.0 g/dL
Hematocrit	25.70%	36.0-46.0%
Mean corpuscular volume	92.4 fL	80.0-100.0 fL
Mean corpuscular hemoglobin	31.2 pg	27.0-33.0 pg
Mean corpuscular hemoglobin concentration	33.8 g/dL	32.0-36.0 g/dL
Red cell distribution width	14.70%	11.5-14.5%
Platelets	110 × 10⁹/L	150-450 × 10⁹/L
Inflammatory markers		
Erythrocyte sedimentation rate	50 mm/hr	0-20 mm/hr
C-reactive protein	28 mg/L	<10 mg/L
Ferritin	30 ng/mL	11-307 ng/mL
Procalcitonin	<0.05 ng/mL	<0.5 ng/mL
Other tests		
Urinalysis	Bacteriuria	None
Hemoglobin A1c	7.80%	<5.7%

The *Lactobacillus* lumbar discitis and osteomyelitis was presumed to originate from a genitourinary source. She was treated with a six-week course of intravenous piperacillin-tazobactam, with a good clinical response, and was subsequently transitioned to oral amoxicillin-clavulanate, completing approximately two months of antibiotic therapy. Her back pain steadily improved, and she was able to ambulate without gait instability. Amoxicillin-clavulanate was eventually discontinued, and she remained well off antibiotics, with no worsening of back pain or new neurological deficits.

## Discussion

*Lactobacillus *species are non-spore-forming, non-obligate, Gram-positive bacillary prokaryotes typically found in mucous membrane-associated tracts in humans [3]. These bacteria are usually considered beneficial in the digestive and reproductive tracts and are used in the management of certain gastrointestinal disorders [4,5].

*L. jensenii *is commonly found in the female genital tract and helps maintain healthy vaginal flora [6]. Certain factors, including sexual intercourse, menstruation, venereal disease, pregnancy, and vaginal irrigation, can disrupt the balance of vaginal flora, potentially leading to bacteremia [6].

Although *L. jensenii *vertebral discitis and osteomyelitis are uncommon, and these bacteria are sometimes regarded as culture contaminants, this case highlights the pathogenic potential of *Lactobacillus *in immunocompromised patients [2]. It underscores the importance for clinicians to recognize the potentially severe consequences and sequelae of disseminated *Lactobacillus *infections if management is delayed [2,5].

In this patient, the *L. jensenii *vertebral osteomyelitis and discitis can be attributed to multiple factors. Her poorly controlled diabetes, combined with pancytopenia, resulted in impaired immunity, increasing susceptibility to invasive infections. Although she had maxillary dentures, she denied recent dental procedures or infections, making a dental source unlikely. The temporal relationship with the resumption of sexual activity after prolonged abstinence strongly supports a genitourinary portal of entry, a mechanism frequently described for *Lactobacillus* bacteremia. This was likely facilitated by the combined effects of poorly controlled diabetes and pancytopenia on her immune system [5,6].

## Conclusions

This report describes an atypical case of vertebral discitis and osteomyelitis caused by *L. jensenii*, which was successfully treated with antibiotics alone, without surgical intervention, and resulted in complete resolution of symptoms. To our knowledge, this represents a rare vertebral and paraspinal infection likely translocated from a genitourinary source, emphasizing the importance of thorough history-taking in identifying unusual infection pathways. Furthermore, this case underscores the pathogenic potential of *Lactobacillus *species, which are typically considered harmless and beneficial components of normal human flora.
